# The splicing mutant of the human tumor suppressor protein DFNA5 induces programmed cell death when expressed in the yeast *Saccharomyces cerevisiae*

**DOI:** 10.3389/fonc.2012.00077

**Published:** 2012-07-25

**Authors:** Sofie Van Rossom, Ken Op de Beeck, Vanessa Franssens, Erwin Swinnen, Anne Schepers, Ruben Ghillebert, Marina Caldara, Guy Van Camp, Joris Winderickx

**Affiliations:** ^1^Department of Biology, Functional Biology, KU LeuvenLeuven-Heverlee, Belgium; ^2^Department of Biomedical Sciences, Center of Medical Genetics, University of AntwerpWilrijk-Antwerp, Belgium

**Keywords:** DFNA5, *Saccharomyces cerevisiae*, yeast, cell death, apoptosis, hearing impairment, tumor suppressor

## Abstract

*DFNA5* was first identified as a gene responsible for autosomal dominant deafness. Different mutations were found, but they all resulted in exon 8 skipping during splicing and premature termination of the protein. Later, it became clear that the protein also has a tumor suppression function and that it can induce apoptosis. Epigenetic silencing of the *DFNA5* gene is associated with different types of cancers, including gastric and colorectal cancers as well as breast tumors. We introduced the wild-type and mutant *DFNA5* allele in the yeast *Saccharomyces cerevisiae*. The expression of the wild-type protein was well tolerated by the yeast cells, although the protein was subject of degradation and often deposited in distinct foci when cells entered the diauxic shift. In contrast, cells had problems to cope with mutant DFNA5 and despite an apparent compensatory reduction in expression levels, the mutant protein still triggered a marked growth defect, which in part can be ascribed to its interaction with mitochondria. Consistently, cells with mutant DFNA5 displayed significantly increased levels of ROS and signs of programmed cell death. The latter occurred independently of the yeast caspase, Mca1, but involved the mitochondrial fission protein, Fis1, the voltage-dependent anion channel protein, Por1 and the mitochondrial adenine nucleotide translocators, Aac1 and Aac3. Recent data proposed DFNA5 toxicity to be associated to a globular domain encoded by exon 2–6. We confirmed these data by showing that expression of solely this domain confers a strong growth phenotype. In addition, we identified a point mutant in this domain that completely abrogated its cytotoxicity in yeast as well as human Human Embryonic Kidney 293T cells (HEK293T). Combined, our data underscore that the yeast system offers a valuable tool to further dissect the apoptotic properties of DFNA5.

## Introduction

Evasion of apoptosis is known to be an important factor in tumorigenesis, but the underlying mechanisms are often not well understood. Therefore, more research is required on the factors that govern cellular decisions between malignant outgrowth or programmed cell death, as this may eventually lead to the design of more efficient anti-cancer therapies (Fulda and Debatin, 2004; Bremer et al., 2006; Call et al., 2008). Here, we describe our findings on the heterologous expression in yeast of a human protein that has an important role in controlling the switch between cell survival and cell death, i.e., DFNA5.

DFNA5 was originally identified in a Dutch family with autosomal dominant non-syndromic hearing impairment (Van Laer et al., 1998). This association with hearing loss was later confirmed with the description of *DFNA5* mutations in a Korean family, two Chinese families and a second Dutch family (Yu et al., 2003; Bischoff et al., 2004; Cheng et al., 2007; Park et al., 2010). Although the mutations in these families are different, they all lead to exon 8 skipping during splicing, thereby causing a frameshift and premature termination of the protein. Another type of mutation was reported in an Iranian family, where an insertion of a cytosine at nucleotide position 640 truncates the protein at a position corresponding to exon 5 of the gene. However, this mutation does not segregate with the hearing loss phenotype and is even present in persons with normal hearing (Van Laer et al., 2007). Hence, it appears that *DFNA5*-associated hearing loss is caused by a gain-of-function mutation due to exon 8 skipping. To date, the exact function of the DFNA5 protein is still unknown, but recent evidence suggests that the protein harbors a pro-apoptotic function (Op de Beeck et al., 2011a). Analysis of the protein revealed that it consists of two globular domains separated by a hinge region, whereby the first domain, consisting of the amino acid residues 1–256, displays pro-apoptotic activity, while the second domain, corresponding to residues 282–496, may serve as a regulator that shields the apoptosis-inducing function of the first domain (Op de Beeck et al., 2011b).

Some studies also associated DFNA5 with several types of cancer, including gastric, colorectal as well as breast cancer. Emerging evidence from the past years suggest that DFNA5 plays a role as a tumor suppressor protein and that the corresponding gene is epigenetically silenced through methylation (Thompson and Weigel, 1998; Lage et al., 2001; Akino et al., 2007; Kim et al., 2008a; Fujikane et al., 2010). Consistent with this role are the observations that siRNA-mediated knock-down of *DFNA5* in non-malignant breast epithelial cell lines enhances colony numbers, colony size and cell growth (Kim et al., 2008b), while forced expression of *DFNA5* in gastric cancer cell lines suppresses colony formation (Akino et al., 2007). Also in support of a tumor suppressor function are the reports that expression of the *DFNA5* gene is controlled by p53 (Masuda et al., 2006) and that silencing of the gene correlates with tumor cell resistance to chemotherapeutic drugs (Lage et al., 2001).

Supported by the observation that the exon 8 splicing mutant of DFNA5 (mutDFNA5), but not the wild-type allele (wtDFNA5), triggers cell cycle arrest when expressed in the fission yeast *Schizosaccharomyces pombe* (Gregan et al., 2003), we decided to use yeast as a model to investigate the role of DFNA5 in more detail. We expressed wtDFNA5 and mutDFNA5 in the budding yeast *Saccharomyces cerevisiae* and analyzed the repercussions on growth, oxidative stress, and the induction of programmed cell death. Data obtained from the wild-type strain and a series of deletion mutants confirmed that mutDFNA5 strongly induces programmed cell death, a phenomenon being dependent on mitochondrial integrity, but independent of the yeast caspase, Mca1. In addition, the yeast model proved to be an ideal tool to identify point mutants in the apoptosis-inducing domain of DFNA5 that abrogated its ability to induce cytotoxicity.

## Materials and methods

### Strains, plasmids, and growth analysis

In this study, we used the BY4741 wild-type strain (Brachmann et al., 1998) and isogenic deletion strains of the genome-wide collection (EUROSCARF, Frankfurt, Germany) lacking proteins involved in programmed cell death as listed in Table 1. The C-terminally HA-tagged full-length wtDFNA5 and mutDFNA5 cDNAs, the C-terminally EGFP fusion proteins and the wtDFNA5 first and second globular fragments were isolated and amplified as previously described (Gregan et al., 2003; Op de Beeck et al., 2011a,b) using the primers listed in Table 2. All amplified products were ligated into the pYX212 plasmid using either *Eco*RI and *Bam*HI or *Eco*RI and *Sal*I restriction sites, thereby placing the inserts under expression control of the constitutive *TPI1* promoter. The mutants HCA-F2R and HCA-A3R at the N-terminal end of the first globular domain were generated by site directed mutagenesis (Agilent, Santa Clara, CA, USA) in combination with the custom designed primers listed in Table 2. All constructs were verified by bidirectional sequencing on an ABI genetic analyser 3130xl (Applied Biosystems, Foster City, CA, USA). The construction of the plasmid for Mito-RFP was previously described (Westermann and Neupert, 2000). Standard transformation techniques were applied (Gietz et al., 1992) and all strains were grown at 30°C in a selective minimal medium containing 2% of glucose (SD-Ura). Growth profiles were determined in 96-well microtiter plates with continuous shaking at 30°C in a Multiskan GO spectrophotometer (Thermo Fisher Scientific Inc., Waltham, MA, USA). Overnight cultures of at least three different transformants were diluted to start new cultures for growth analysis. Growth was monitored until the stationary phase was reached. Growth curves are depicted with scaled OD units and as the mean values of the transformants, with error bars representing standard deviations. The growth profiles of the strains expressing HA- or EGFP-tagged wtDNFNA5 or mutDFNA5 were compared to that of a control strain transformed with either empty vector or a plasmid allowing for expression of EGFP. The differences in time required to reach half maximal optical densities (Δ T1/2) were calculated and used as standards for growth quantification. The difference in Δ T1/2 obtained for wtDFNA5 (ΔT1/2 wtDFNA5-Control) and mutDFNA5 (ΔT1/2 mutDFNA5-Control) in the BY4741 wild-type strain was used as reference and set as 100%.

**Table 1:** **Strain list**.

**Strain**	**Genotype**	**Reference/Source**
BY 4741	Mat**a** *his3*Δ*1 leu2*Δ*0*	Brachmann et al., 1998
	*met15*Δ*0 ura3*Δ*0*	
*aac1*Δ	BY4741*YMR056c*::kanMX4	EUROSCARF
*aac3*Δ	BY4741*YBR085w*::kanMX4	EUROSCARF
*aif1*Δ	BY4741*YNR074c*::kanMX4	EUROSCARF
*dnm1*Δ	BY4741*YLL001w*::kanMX4	EUROSCARF
*fis1*Δ	BY4741*YIL065c*::kanMX4	EUROSCARF
*mca1*Δ	BY4741*YOR197w*::kanMX4	EUROSCARF
*mdv1*Δ	BY4741*YJL112w*::kanMX4	EUROSCARF
*nma111*Δ	BY4741*YNL123w*::kanMX4	EUROSCARF
*nuc1*Δ	BY4741*YJL208c*::kanMX4	EUROSCARF
*por1*Δ	BY4741*YNL055c*::kanMX4	EUROSCARF
*por2*Δ	BY4741*YIL114c*::kanMX4	EUROSCARF
*rny1*Δ	BY4741*YPL123c*::kanMX4	EUROSCARF
*snl1*Δ	BY4741*YIL016w*::kanMX4	EUROSCARF
*tdh2*Δ	BY4741*YJR009c*::kanMX4	EUROSCARF
*tdh3*Δ	BY4741*YGR192c*::kanMX4	EUROSCARF
*tim18*Δ	BY4741*YOR297c*::kanMX4	EUROSCARF
*ymr074c*Δ	BY4741*YMR074c*::kanMX4	EUROSCARF
*ysp1*Δ	BY4741*YHR155w*::kanMX4	EUROSCARF

**Table 2:** **Primer pairs**.

**Construct**	**Forward primer**	**Reverse primer**
wtDFNA5-HA	TATATATATAGAATTCATGTTTGCCAAAGCAACCAGGAATT	GACCGGTGGATCCCGTGAATGTTCTCTGCCTAAAGC
mutDFNA5-HA	TATATATATAGAATTCATGTTTGCCAAAGCAACCAGGAATT	GACCGGTGGATCCCGAGGTTGGGTCTTCAAGATCAG
wtDFNA5-EGFP	TATATATATAGAATTCATGTTTGCCAAAGCAACCAGGAATT	TATATATAGTCGACGCTTGTACAGCTCGTCCATGCC
mutDFNA5-EGFP	TATATATATAGAATTCATGTTTGCCAAAGCAACCAGGAATT	TATATATAGTCGACGCTTGTACAGCTCGTCCATGCC
DFNA5 domain A	TATATATATAGAATTCATGTTTGCCAAAGCAACCAGGAATT	CGGTGGATCCCGGTCCAGGTAGACAGAGTCAAT
DFNA5 domain B	AAGCTTCGAATTCTGGCCACCATGGACCCCCTGGTCTTTCGAGAG	GACCGGTGGATCCCGTGAATGTTCTCTGCCTAAAGC
HCA-F2R mutant^*^	GAGCTCAAGCTTCGAATTCTGATGCGTGCCAAAGCAACCAGG	CCTGGTTGCTTTGGCACGCATCAGAATTCGAAGCTTGAGCTCG
HCA-A3R mutant^*^	CGAGCTCAAGCTTCGAATTCTGATGTTTCGTAAAGCAACCAGG	CCTGGTTGCTTTACGAAACATCAGAATTCGAAGCTTGAGCTCG

Restriction sites used for cloning are underlined.

* Site directed mutagenesis primers.

Human Embryonic Kidney 293T cells (HEK293T) were subcultured in 60 mm dishes at a density of 2 × 10^6^ cells in Dulbecco's modified Eagle's medium containing 4500 mg/l glucose supplemented with 10% (v/v) fetal calf serum, 100 U/ml penicillin, 100 μg/ml streptomycin and 2 mM L-glutamine (all products from Invitrogen, San Diego, CA, USA). The cells were incubated overnight at 37°C in a 5% CO_2_ humidified environment. The plasmids used for wtDFNA5 and mutDFNA5 expression in mammalian HEK293T cells, after transfection with lipofectamine, were described before (Op de Beeck et al., 2011b). In addition, we used the pEGFP-N1 vector to construct plasmids for the expression of HCA-F2R and HCA-A3R mutants.

### Western blot analysis

Yeast samples were grown in 3 ml cultures on selective medium and were harvested at an OD_600 nm_ between 1.5 and 2.0. An equal amount of cells were taken and lysed by boiling for 15 min in SDS sample buffer [50 mM Tris (pH 8.0), 10 mM β-mercaptoethanol, 2% SDS, 0.1% bromophenol blue, 10% glycerol]. Proteins were separated by standard SDS-PAGE and blotted onto PVDF membranes (Immobilon-P transfer membranes, Millipore, MA, USA). For immunodetection we used the primary rabbit anti-HA or anti-EGFP antibodies (Santa Cruz, CA, USA) and a secondary horse radish peroxidase (HRP) conjugated goat anti-rabbit antibody (Santa Cruz, CA, USA). The endogenous yeast alcohol dehydrogenase Adh2 served as internal standard. Membranes were developed using the ECL detection kit (Thermo Scientific, IL, USA).

### Flow cytometric analysis of cell death, ROS accumulation and caspase activity

Tests for apoptotic and necrotic markers, using AnnexinV/Propidium Iodide (AV/PI) co-staining, as well as ROS accumulation, using the superoxide-driven conversion of non-fluorescent dihydroethidium (DHE) to fluorescent ethidium, were performed and quantified using BD Influx flow cytometry (BD Biosciences, New Jersey, NJ, USA) as described previously (Büttner et al., 2008). Yeast samples were harvested at different time points. Samples were collected at mid-exponential phase at an OD_600 nm_ between 3.5 and 4.0, just after cells had traversed the diauxic shift (PD) at an OD_600 nm_ between 6.0 and 7.0, and in stationary phase (ST) at an OD_600 nm_ of 8.5 or higher. Analysis of the BD influx flow cytometry data was performed using the software program FlowJo (Tree Star Inc., Ashland, MA, USA). Viability tests of the HCA-F2R and HCA-A3R mutants in HEK293T cells were performed on a FACScan flow cytometer (BD Biosciences, New Jersey, NJ, USA) after staining of the cells with PI. Cell viability was then determined as the ratio of cells showing no PI fluorescence to the total cell population.

A previously described protocol was used to measure the caspase activity (Madeo et al., 2002). Yeast cells grown on selective medium were harvested at an OD_600 nm_ of approximately 4.5. A staining solution containing 10 μM FITC-VAD-FMK in PBS (CaspACE, Promega, WI, USA) was added to an amount of cells corresponding to an OD_600 nm_ of 0.5 and incubated for 20 min at room temperature. After washing and resuspension in 200 μl PBS flow cytometric analysis was performed using a 530/40 bandpass filter.

### Fluorescence microscopy

Cells transformed with wtDFNA5 or mutDFNA5 fused to EGFP were grown till mid-exponential or post-diauxic phase as indicated and visualized using a Leica DM4000B fluorescence microscope (Leica Microsystems GmbH, Wetzlar, Germany). Pictures were taken with a Leica DFC420C camera using the Leica Application Suite software. The percentages of post-diauxic cells with or without inclusions were determined by manual counting of at least 300 cells per sample.

Mitochondria were visualized by the expression of a mitochondria-targeted red fluorescent protein, Mito-RFP (Westermann and Neupert, 2000). To stain the vacuolar membrane, cells were in the post-diauxic phase and incubated with FM4-64 ([N-(3-triethylammoniumpropyl)-4-(p-diethylamino phenylhexa-trienyl) pyridinium dibromide]; Molecular Probes, Eugene, OR, USA) at room temperature for at least 30 min in a HEPES buffer containing 1% of glucose to facilitate the uptake of FM4-64. To visualize the nucleus, we performed a 4',6-diamidino-2-phenylindole (DAPI) staining. The cells were harvested in the post-diauxic phase and incubated for 20 min in a phosphate buffer (0.04 M K_2_HPO_4_, 0.01 M KH_2_PO_4_, 0.15 M NaCl, 0.1 g/100 ml NaN_3_) containing 50% ethanol. After washing with PBS, DAPI was added (1 μg/μl) and samples were incubated at room temperature for 15 min.

### Statistical analysis

All experiments included biological replicates and the use of independent transformants. Statistical analysis was performed using unpaired *t*-tests or One-Way ANOVA.

## Results

### Mutant DFNA5 induces apoptotic and necrotic cell death in yeast

**Figure 1:**
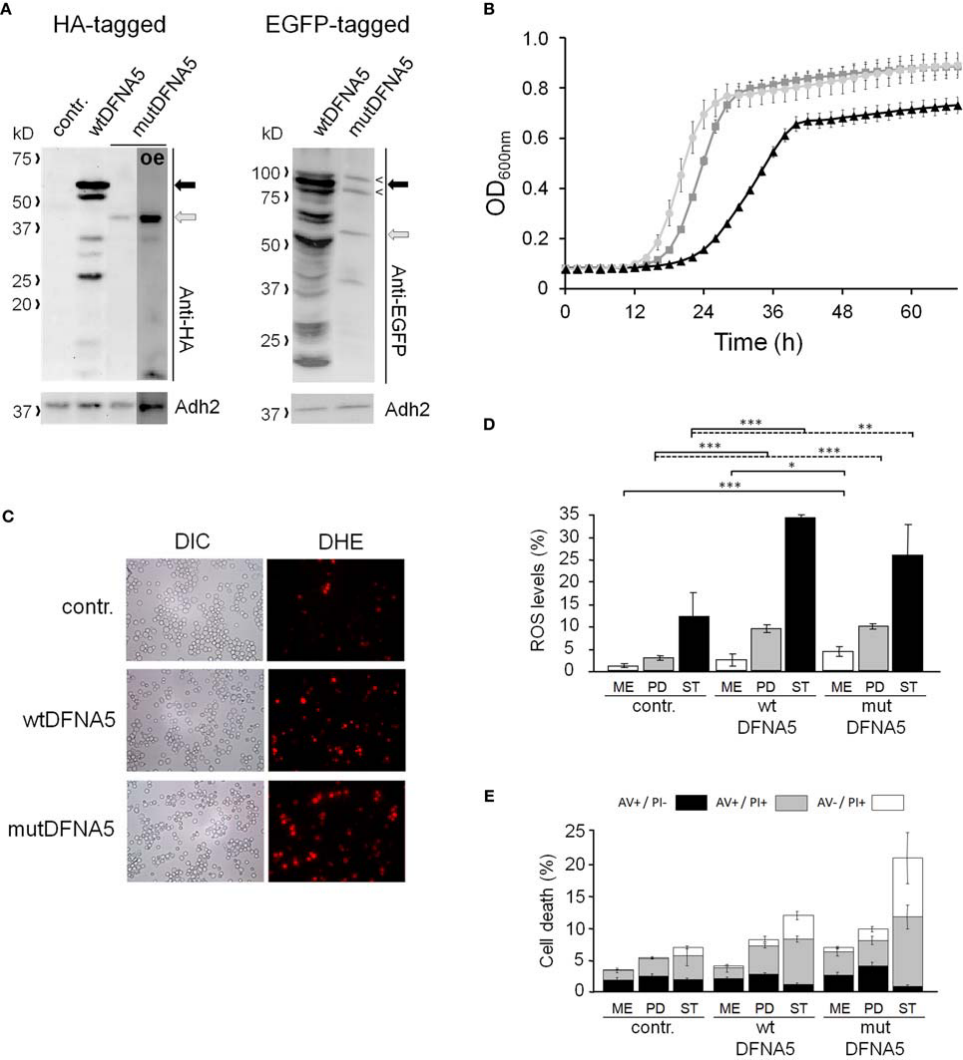
**Repercussions of wtDFNA5 and mutDFNA5 expression in BY4741 wild-type cells**. **(A)** Western blot analysis of protein extracts of the BY4741 wild-type strain transformed with an empty plasmid (contr.) or constructs allowing for the expression of C-terminally HA-tagged and EGFP-tagged wtDFNA5 or mutDFNA5 as indicated. Immunodetection was performed using primary antibodies directed against the HA-tag, EGFP, or Adh2, which was used as loading control. The black arrow indicates full-length wtDFNA5 and the gray arrow full-length mutDFNA5. The small open arrowheads indicate a-specific bands detected by anti-EGFP. For mutDFNA5, the lane marked with [oe] represents an overexposure of the Western blot. **(B)** Growth of the BY4741 wild-type strain transformed with an empty plasmid (light gray circles) or a construct allowing for expression of C-terminally HA-tagged wtDFNA5 (dark gray squares) or mutDFNA5 (black triangles). **(C)** Visualization of ROS producing cells in control cultures (contr.) or cultures of cells expressing C-terminally HA-tagged wtDFNA5 or mutDFNA5 in post-diauxic phase. **(D)** Quantification of ROS accumulation using DHE staining in control cultures (contr.) or cultures of cells expressing C-terminally HA-tagged wtDFNA5 or mutDFNA5 when sampled at the mid-exponential growth phase (ME), at post-diauxic shift (PD), and in stationary phase (ST). **(E)** Quantification of the number of AV/PI positive cells in ME, PD, and ST phase of control cells or cells expressing HA-tagged wtDFNA5 or mutDFNA5. Significances were assayed using unpaired *t-*tests. For the AV/PI co-staining the following significances were obtained when compared to the control: for wtDFNA5 in ME: AV^−^/PI^+ *^; for wtDFNA5 in PD: AV^+^/PI^+ ***^, AV^−^/PI^+ *^; for wtDFNA5 in ST: AV^+^/PI^− *^, AV^+^/PI^+ *^, AV^−^/PI^+ **^; for mutDFNA5 in ME: AV^+^/PI^− *^, AV^+^/PI^+ **^, AV^−^/PI^+ **^; for mutDFNA5 in PD: AV^+^/PI^− **^, AV^+^/PI^+ *^, AV^−^/PI^+ ***^; for mutDFNA5 in ST AV^+^/PI^− **^, AV^+^/PI^+ **^, AV^−^/PI^+ *^. (^*^ = *p* < 0.05; ^**^ = *p* < 0.01; ^***^ = *p* < 0.001).

To study the properties of human DFNA5 in a well-defined model, we expressed the cDNAs of wtDFNA5 and mutDFNA5 in the BY4741 wild-type strain. We used high-copy-number plasmids allowing expression of wtDFNA5 and mutDFNA5 as C-terminally HA-tagged proteins under the control of the strong constitutive *TPI1* promoter. For wtDFNA5, this resulted in good expression levels of the full-length protein, though we noticed that the protein was subject of proteolytic degradation as evidenced by the presence of discrete breakdown products upon Western blot analysis (Figure 1A). A lower expression level was obtained for mutDFNA5 and, interestingly, no major proteolytic fragments were observed in this case, even not when higher concentrations of protein extracts were loaded or when the exposure time of the immunoblots was increased. Growth analysis of the transformants revealed that the expression of wtDFNA5 had only a moderate effect on growth, while expression of mutDFNA5 triggered a significant growth defect. The latter was characterized by a longer doubling time and a lower maximal optical density of the cultures (Figure 1B). Hence, as compared to a culture of the BY4741 strain transformed with the empty plasmid, a culture of cells expressing wtDFNA5 required on average only an additional 3 h (SD ± 0.24) to reach half of the maximal optical density (Δ T1/2), whereas for a culture of cells expressing mutDFNA5 this Δ T1/2 was extended to about 11.5 h (SD ± 0.32) (Table 3). Combined these data suggest that yeast cells tolerated the presence of wtDFNA5 fairly well but have problems to cope with mutDFNA5. Notably, since the mutant protein seemed to escape the protein breakdown, the cells apparently counter selected and reduced the expression of the mutant protein to prevent extreme harmful effects.

**Table 3:** Quantification of growth^*^.

**Strain**	**Function**	**T1/2 Control**	**T1/2 wtDFNA5**	**T1/2 mutDFNA5**	**ΔT1/2 wtDFNA5-Control**	**ΔT1/2 mutDFNA5-Control**	**%ΔT1/2 mutDFNA5-wtDFNA5**
BY4741 (HA-tagged)		19.83 ± 0.52	22.83 ± 0.26	31.38 ± 0.48	3.00 ± 0.24	11.54 ± 0.32	100.00 ± 1.42
BY4741 (EGFP-tagged)		20.30 ± 1.15	24.90 ± 2.61	35.88 ± 3.54	4.60 ± 1.27	15.58 ± 1.84	128.49 ± 8.60
*aac1*Δ	Mitochondrial ADP/ATP Carrier	31.30 ± 1.20	28.13 ± 5.36	31.08 ± 1.36	−3.18 ± 2.73	−0.22 ± 0.77	34.63 ± 11.17
*aac3*Δ	Mitochondrial ADP/ATP Carrier	24.70 ± 0.45	27.00 ± 0.82	29.40 ± 1.67	2.30 ± 0.45	4.70 ± 0.77	28.10 ± 3.37
*aif1*Δ	Mitochondrial Apoptosis-inducing factor	19.60 ± 0.22	23.13 ± 0.25	33.00 ± 0.82	3.53 ± 0.16	13.40 ± 0.42	115.61 ± 1.76
*dnm1*Δ	Dynamin-related GTPase, mitochondrial fission	20.20 ± 0.27	22.33 ± 1.61	31.50 ± 2.38	2.13 ± 0.94	11.30 ± 1.20	107.32 ± 6.07
*fis1*Δ	Mitochondrial membrane fission	26.70 ± 1.10	34.38 ± 1.80	48.67 ± 1.15	7.68 ± 1.02	21.97 ± 0.83	167.32 ± 5.26
*mca1*Δ	Putative cysteine protease, metacaspase	21.80 ± 0.27	24.30 ± 0.45	34.50 ± 2.27	2.50 ± 0.23	12.70 ± 1.14	119.41 ± 4.54
*mdv1*Δ	Dmn1 adaptor protein, mitochondrial fission	20.40 ± 1.34	24.38 ± 0.25	31.90 ± 2.01	3.98 ± 0.61	11.50 ± 1.08	88.10 ± 4.66
*nma111*Δ	Omi1/HtrA2 Ortholog, Serine protease	20.00 ± 0.00	23.67 ± 0.58	31.25 ± 2.22	3.67 ± 0.33	11.25 ± 1.11	88.78 ± 4.82
*nuc1*Δ	Mitochondrial nuclease, Endo G ortholog	22.38 ± 2.14	24.10 ± 0.55	33.67 ± 0.58	1.73 ± 1.10	11.29 ± 1.12	112.00 ± 6.54
*por1*Δ	Mitochondrial porin, VDAC homolog	20.50 ± 1.50	25.40 ± 0.65	40.00 ± 1.38	4.90 ± 0.91	19.50 ± 1.03	170.93 ± 5.53
*por2*Δ	Putative mitochondrial porin, VDAC homolog	21.20 ± 0.45	23.90 ± 0.42	31.30 ± 2.49	2.70 ± 0.27	10.10 ± 1.13	86.63 ± 4.31
*rny1*Δ	Ribonuclease from yeast	20.90 ± 0.55	22.80 ± 0.57	33.90 ± 2.13	1.90 ± 0.35	13.00 ± 0.98	129.95 ± 3.87
*snl1*Δ	Suppressor of nup116-C lethal, Bag-1 homolog	18.60 ± 0.89	20.38 ± 0.25	28.00 ± 0.00	1.78 ± 0.42	9.40 ± 0.40	89.27 ± 2.33
*tdh2*Δ	Triose-phosphate dehydrogenase	20.13 ± 0.25	23.00 ± 0.50	31.00 ± 1.54	2.88 ± 0.31	10.88 ± 0.70	93.66 ± 3.07
*tdh3*Δ	Triose-phosphate dehydrogenase	22.00 ± 0.00	23.88 ± 0.25	35.50 ± 1.00	1.88 ± 0.13	13.50 ± 0.50	136.10 ± 2.13
*tim18*Δ	Translocase of the inner mitochondrial membrane	21.40 ± 0.42	25.50 ± 0.50	33.50 ± 0.58	4.10 ± 0.34	12.10 ± 0.34	93.66 ± 1.96
*ymr074c*Δ	Protein with homology to human PDCD5	20.75 ± 0.50	23.70 ± 0.57	29.00 ± 3.32	2.95 ± 0.36	8.25 ± 1.50	62.05 ± 6.03
*ysp*Δ	Yeast suicide protein	20.00 ± 0.00	23.40 ± 1.39	34.10 ± 1.95	3.40 ± 0.62	14.10 ± 0.87	125.27 ± 4.18

T1/2: time to reach half maximal optical density (hours).

Δ*T1/2: difference in time to reach half maximal optical density (hours).*

* Values are expressed as mean ± standard deviation.

It is known that cytotoxic effects instigated by heterologous proteins are often a reflection of a failing protein quality control and clearance system, which then leads to enhanced oxidative stress and eventually increased cell death [reviewed in Winderickx et al., 2008]. To examine whether this is also the case for heterologous expression of DFNA5, we measured the level of reactive oxygen species (ROS) using a DHE staining on culture samples taken at different time points during growth (Figures 1C,D). In mid-exponential cultures, the ROS levels were only significantly increased in cells expressing mutDFNA5 when compared to the control. However, once the cultures traversed the diauxic shift and switched their metabolism to respiration, a marked increment of the ROS level was observed for both the cells expressing wtDFNA5 and those expressing mutDFNA5. In case of wtDFNA5, the level of ROS in the early post-diauxic phase was about three times higher than that of the control cells and it further increased in stationary phase. With mutDFNA5, the increment in ROS in the early post-diauxic phase was comparable to wtDFNA5, though by the time these cells reached the stationary phase the average ROS level was lower than that of cells expressing wtDFNA5.

We also performed a co-staining with AV/PI to detect cells showing signs of apoptotic and necrotic cell death (Figure 1E). This again revealed that only the expression of mutDFNA5 significantly enhanced cell death during the mid-exponential phase, while both expression of wtDFNA5 or mutDFNA5 enhanced cell death once the cultures were beyond the diauxic shift, albeit to a different extent. More in particular, we noticed that in the post-diauxic and stationary phase the expression of the wild-type and especially the mutant allele triggered an increase in the number of late apoptotic (AV^+^/PI^+^) and necrotic cells (AV^−^/PI^+^) and that in the stationary phase this was even associated with a significant decrease in early apoptotic (AV^+^/PI^−^) cells. That the increments of late apoptotic and necrotic cells are most pronounced upon expression of mutDFNA5 is consistent with the observed enhanced growth defect. Furthermore, these results are also in agreement with previously reported observations of enhanced apoptotic and necrotic cell death in mammalian cells transfected with mutDFNA5 (Van Laer et al., 2004; Op de Beeck et al., 2011b).

### Mutant DFNA5 escapes protein quality control deposition and interacts with mitochondria

To analyse the intracellular localization, we expressed wtDFNA5 and mutDFNA5 as a C-terminally tagged EGFP fusion. Their relative expression levels were comparable to those of the HA-tagged counterparts. Furthermore, similar as for the HA-tagged versions, the wtDFNA5-EGFP fusion was subject to proteolytic degradation, while this was by far less pronounced for the mutDFNA5-EGFP fusion (Figure 1A). Despite of these similarities, we noticed that the fusion proteins induced slightly enhanced growth defects as judged from the Δ T1/2 values calculated based on the growth difference with control cultures expressing native EGFP (Δ T1/2 wtDFNA5-EGFP: 4.60 h ± 1.27; Δ T1/2 mutDFNA5-EGFP: 15.85 h ± 1.84; Table 3). Nonetheless, since also in this case mutDFNA5-EGFP instigated a much higher cytotoxicity than wtDFNA5-EGFP, we reasoned that further analysis would still provide important insight in the differential properties of the proteins.

**Figure 2:**
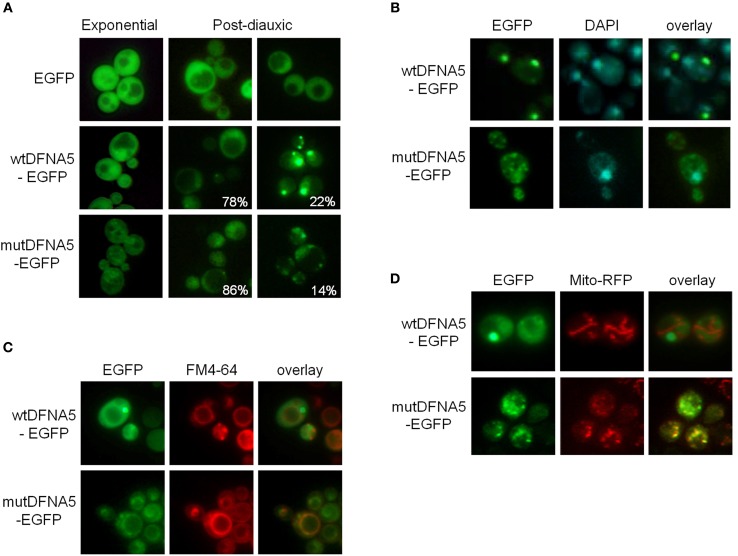
**Wild-type and mutant DFNA5 form inclusions in yeast**. **(A)** Fluorescence microscopic visualization and intracellular localization of wtDFNA5-EGFP and mutDFNA5-EGFP fusion proteins in the BY4741 wild-type yeast strain sampled at the mid-exponential growth phase or in the post-diauxic growth phase as indicated. Percentages refer to the number of cells displaying a dispersed cytoplasmic localization or with inclusions. Cells expressing only EGFP served as control. **(B,C)** Pictures of post-diauxic wild-type cells with inclusions formed by wtDFNA5-EGFP or mutDFNA5-EGFP and stained with DAPI (panel **B**) to visualize the nucleus or with FM4-64 (panel **C**) to visualize the vacuolar membrane. **(D)** Pictures of post-diauxic wild-type cells expressing wtDFNA5-EGFP or mutDFNA5-EGFP and a mitochondrial red fluorescent marker protein, Mito-RFP.

Fluorescence microscopy confirmed the difference in expression level between wtDFNA5-EGFP and mutDFNA5-EGFP. It also showed that wtDFNA5-EGFP was evenly distributed over the cytoplasm in mid-exponential growth phase, although we noticed that about one fifth of the cells (22%) gradually accumulated fluorescent material in more dense inclusions (Figure 2A). This resulted in the formation of one or a few distinct deposits by the time these cells reached the post-diauxic phase. Similar as in transfected mammalian cells (Van Laer et al., 2004), the distribution of mutDFNA5-EGFP in mid-exponential phase cells appeared to be more granulated and possibly confined to intracellular structures, though it was difficult to assign a definite pattern due to the low expression level of the fusion protein. In the post-diauxic phase, inclusions were present in about one out of seven cells that expressed mutDFNA5-EGFP (14%). As compared to the deposits of wtDFNA5-EGFP, the inclusions formed by the mutant protein were usually less intense and occurring as small foci in the vicinity of the plasma membrane (Figure 2A).

It was previously shown that cells protect themselves by sequestering breakdown products and damaged or aggregated proteins in different protein quality control compartments, referred to as aggresomes or JUNQ and IPOD. JUNQ represents a juxta-nuclear quality control compartment that serves as a temporary storage site for misfolded proteins, keeping them in an ubiquitinated, soluble state for either refolding or degradation by the ubiquitin-proteasome system. IPOD, on the other hand, is a perivacuolar compartment for the deposit of insoluble, non-ubiquitinated substrates, such as amyloidic proteins, that possibly await clearance by means of autophagy (Bagola and Sommer, 2008; Kaganovich et al., 2008). To analyse in more detail the localization of the inclusions formed by wtDFNA5-EGFP and mutDFNA5-EGFP, we performed stainings with DAPI and FM4-64 to, respectively, visualize the nucleus and the vacuolar membrane. This demonstrated that the larger deposits of wtDFNA5-EGFP did not co-localize with the nucleus (Figure 2B). Instead, these deposits were found at the periphery of the vacuole and thus are likely to correspond to IPOD (Figure 2C). The small foci formed by mutDFNA5-EGFP, neither co-localized with the nucleus, nor with the vacuolar membrane but, interestingly, seemed to partially overlap with DAPI-stained mitochondrial DNA. This led us to visualize the mitochondrial network. The strains were therefore co-transformed with a plasmid enabling the expression of a mitochondrial red fluorescent marker protein, Mito-RFP (Westermann and Neupert, 2000). Further analysis revealed that, indeed, the small foci of mutDFNA5-EGFP often co-localized with punctuated fragmented mitochondria and this in contrast to the larger deposits of wtDFNA5-EGFP (Figure 2D).

### Mutant DFNA5 induces cell death independently of caspase

**Figure 3:**
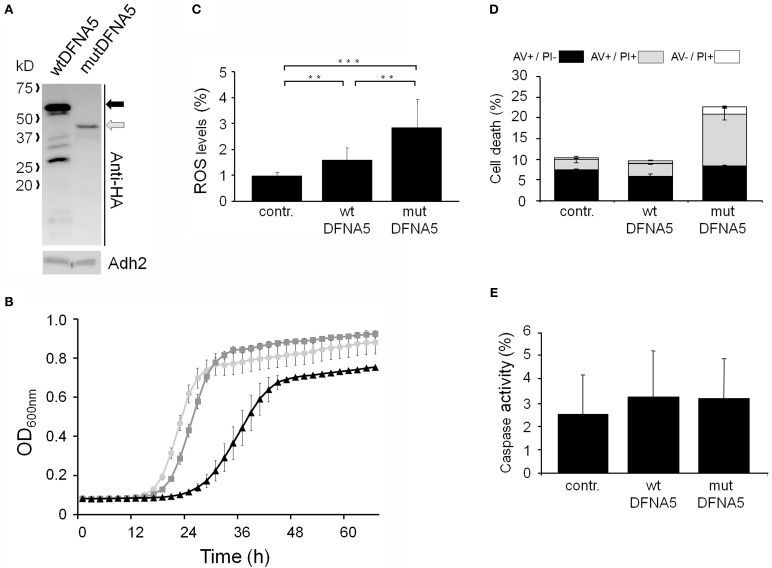
**mutDFNA5-induced cell death does not involve the yeast caspase**. **(A)** Western blot analysis of protein extracts of *mca1*Δ cells transformed with constructs allowing for the expression of C-terminally HA-tagged wtDFNA5 or mutDFNA5 as indicated. Immunodetection was performed using primary antibodies directed against the HA-tag or Adh2, which was used as loading control. The black arrow indicates full-length wtDFNA5 and the gray arrow full-length mutDFNA5. **(B)** Growth profiles of *mca1*Δ cells expressing HA-tagged wtDFNA5 (dark gray squares) or mutDFNA5 (black triangles) or transformed with an empty plasmid (light gray circles). **(C,D)** Quantification of ROS accumulation using DHE staining (panel **C**) or AV/PI positive cells (panel **D**) in mid-exponential cultures of *mca1*Δ cells transformed with an empty plasmid (contr.) or with constructs allowing for expression of C-terminally HA-tagged wtDFNA5 or mutDFNA5. **(E)** Assessment of the caspase activity using FITC-tagged z-VAD-FMK in BY4741 wild-type cells expressing C-terminally HA-tagged wtDFNA5 or mutDFNA5 or transformed with an empty plasmid (contr.). Significances were assayed using unpaired *t*-tests. For the AV/PI co-staining the following significances were obtained when making the comparison with the control: wtDFNA5: AV^−^/PI^+ *^; mutDFNA5: AV^+^/PI^+ ***^, AV^−^/PI^+ ***^. (^*^ = *p* < 0.05; ^**^ = *p* < 0.01; ^***^ = *p* < 0.001).

Next, we systematically assessed the repercussion of wtDFNA5 and mutDFNA5 expression in strains harboring deletions of key players of the programmed cell death machinery. We monitored the expression of the HA-tagged DFNA5 proteins, compared the growth profiles and measured the levels of ROS and cell death during mid-exponential growth. One of the strains analysed was the mutant lacking the yeast caspase, Mca1, which allowed us to establish whether DFNA5-induced cell death involves the previously described caspase-dependent or caspase-independent processes (Madeo et al., 2009). As compared to the corresponding wild-type strains, the *mca1*Δ strains displayed similar expression profiles of wtDFNA5 and mutDFNA5 (Figure 3A) and albeit the mutant strains grew more slowly, they maintained similar DFNA5-dependent growth defects as determined by calculating the Δ T1/2 values (Table 3 and Figure 3B). In addition, the deletion of *MCA1* did not prevent accumulation of ROS upon expression of mutDFNA5, nor did it prevent the mutDFNA5-instigated induction of apoptotic and necrotic cell death markers (Figures 3C,D). Consistently, treatment of BY4741 wild-type cells with the FITC-labeled pancaspase inhibitor z-VAD-FMK, which binds to the active site of caspases in yeast (Madeo et al., 2002), did not provide evidence for enhanced caspase activity upon the expression of wtDFNA5 or mutDFNA5 (Figure 3E). When combined, these observations suggest that mutDFNA5-induced cell death occurs mainly independent of the caspase Mca1. Likewise, we could exclude several of the other known players of the yeast apoptotic machinery to have a major impact on the DFNA5-dependent phenotypes. These included the mitochondrial cell death effector, Aif1, the mitochondrial endonuclease G, Nuc1, the ortholog of the mammalian Omi/HtrA2 serine protease, Nma111, as well as the yeast suicide protein, Ysp2 (Table 3 and data not shown).

### Mutant DFNA5 induces cell death through mitochondrial functions

**Figure 4:**
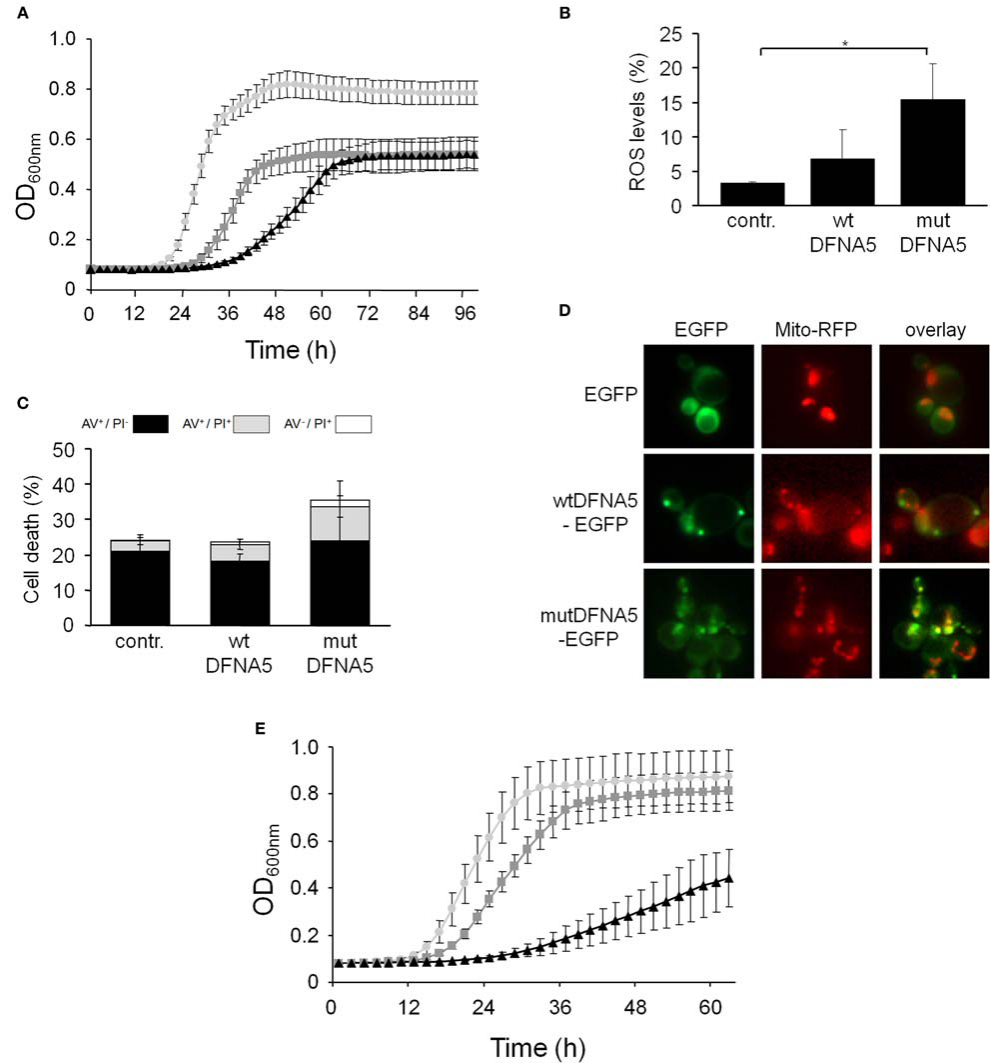
**DFNA5 cytotoxicity is enhanced in cells lacking mitochondrial functions**. **(A)** Growth profiles of *fis1*Δ cells expressing C-terminally HA-tagged wtDFNA5 (dark gray squares) or mutDFNA5 (black triangles) or transformed with an empty plasmid (light gray circles). **(B,C)** Quantification of ROS accumulation using DHE staining (panel **B**) or AV/PI positive cells (panel **C**) in mid-exponential *fis1*Δ cultures without (contr.) or with expression of HA-tagged wtDFNA5 or mutDFNA5. **(D)** Fluorescence microscopy pictures of *fis1*Δ cells expressing EGFP or displaying inclusions of wtDFNA5-EGFP or mutDFNA5-EGFP and co-transformed with Mito-RFP to visualize mitochondria. **(E)** Growth profiles of *por1*Δ cells expressing HA-tagged wtDFNA5 (dark gray squares) or mutDFNA5 (black triangles) or transformed with an empty plasmid (light gray circles). Significances were assayed using unpaired *t-*tests. For the AV/PI co-staining of the *fis1*Δ cultures the following significant *p*-values were obtained when making the comparison with the control and mutDFNA5: AV^+^/PI^+ **^. (^*^ = *p* < 0.05; ^**^ = *p* < 0.001).

In contrast to the mutant strains mentioned above, we found increased DFNA5-induced cytotoxicity in the strains lacking either the mitochondrial outer membrane protein, Fis1 or the voltage-dependent anion channel protein, Por1. The Fis1 protein is involved in mitochondrial fission that attracts the dynamin-related GTPase, Dnm1 through the adaptors Mdv1 and Caf4. The complex then forms a contractile ring that promotes outer membrane division. Interesting is that with the *fis1*Δ strains, both the cultures expressing wtDFNA5 or mutDFNA5 displayed a comparable lower maximal optical density (Figure 4A). This is similar to what we observed for cultures of wild-type cells transformed with mutDFNA5 and it probably reflects the disturbance of mitochondrial dynamics. The role of Fis1 and mitochondrial fission in programmed cell death is still not fully clarified and seems to depend on the type of the cell death stimulus (Braun and Westermann, 2011). For instance, for ethanol-induced apoptosis, Fis1 was shown to mediate mitochondrial fragmentation and cell death independently of Dnm1 and Mdv1 (Kitagaki et al., 2007), whereas for acetic acid-induced apoptosis, Fis1 was reported to protect cells by inhibition of Dnm1- and Mdv1-mediated mitochondrial fission and cell death (Fannjiang et al., 2004). Concerning the DFNA5-induced cell death, Fis1 obviously exerted a protective function, but this appeared to be largely independent of Dnm1 and Mdv1 because neither the deletion of *DNM1*, nor the deletion of *MDV1* affected the DFNA5-mediated growth phenotype (Table 3). As was to be expected, the levels of ROS were generally higher in the *fis1*Δ strains than in the wild-type strains with a minor increment in case of wtDFNA5 expression, but a clear augmentation in case of mutDFNA5 expression (Figure 4B). Likewise, the amount of dying cells in mid-exponential cultures were in general higher in the *fis1*Δ strains as compared to the wild-type strains, especially the number of early apoptotic cells, and while there was no significant increase in the total number of cells showing signs of cell death between the control and cultures expressing wtDFNA5 or mutDFNA5, the latter two still showed a trend toward enhanced late apoptosis and necrosis (Figure 4C). Analysis of *fis1*Δ cells with combined expression of Mito-RFP and EGFP-fusions showed that most cells contained fragmented and aggregated mitochondria, which did not overlap with the deposits formed by wtDFNA5-EGFP but clearly co-localized with the foci of mutDFNA5-EGFP (Figure 4D). Similar as the loss of Fis1, also the absence of the channel protein Por1 appeared to sensitize cells for DFNA5-mediated cell death. However, with this *por1*Δ deletion strain it was difficult to correctly assess the repercussions on ROS accumulation or cell death due to the very slow growth of the cells transformed with mutDFNA5 (Figure 4E).

**Figure 5:**
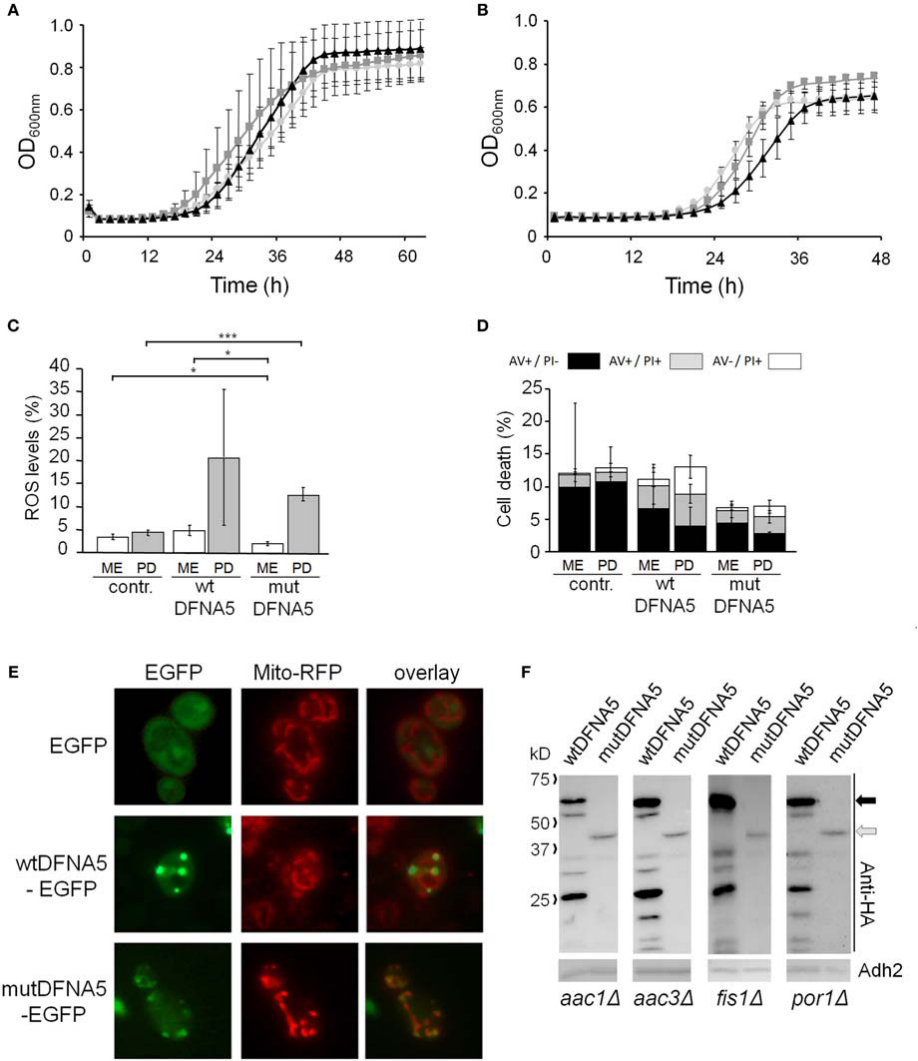
**mutDFNA5 cytotoxicity depends on the mitochondrial ADP/ATP carriers**. **(A,B)** Growth profiles of **(A)** *aac1*Δ cells or **(B)** *aac3*Δ cells expressing C-terminally HA-tagged wtDFNA5 (dark gray squares) or mutDFNA5 (black triangles) or transformed with an empty plasmid (light gray circles). **(C,D)** Quantification of ROS accumulation using DHE staining (panel **C**) or AV/PI positive cells (panel **D**) in mid-exponential (ME) and post-diauxic (PD) phase in *aac3*Δ cultures without (contr.) or with expression of HA-tagged wtDFNA5 or mutDFNA5. **(E)** Fluorescence microscopy pictures of *aac3*Δ cells expressing EGFP or displaying inclusions of wtDFNA5-EGFP or mutDFNA5-EGFP and co-transformed with Mito-RFP to visualize mitochondria. **(F)** Western blot analysis of protein extracts of the *aac1*Δ, *aac3*Δ, *fis1*Δ, *por1*Δ mutant strains transformed with constructs allowing for the expression of C-terminally HA-tagged wtDFNA5 or mutDFNA5 as indicated. Immunodetection was performed using primary antibodies directed against the HA-tag or Adh2, which was used as loading control. Significances were assayed using unpaired *t-*tests. For the AV/PI co-staining the following significances were obtained when compared to the control: for wtDFNA5 in PD: AV^+^/PI^+ *^, AV^−^/PI^+ *^. (^*^ = *p* < 0.05; ^***^ = *p* < 0.001).

In two other mitochondrial mutants, i.e., the *aac1*Δ and *aac3*Δ deletion strains (Figures 5A,B), the growth differences between the control cultures and the cultures expressing wtDFNA5 or mutDFNA5 were almost annihilated, indicative that the lack of Aac1 or Aac3 abrogated the mutDFNA5-associated cytotoxicity. *AAC1* and *AAC3* encode for two of the three ADP/ATP carriers of the inner mitochondrial membrane. Previously reported studies implicated these proteins as effectors of acetic acid-induced apoptosis, a role which apparently does not depend on their ADP/ATP translocase activity but rather on their impact on the mitochondrial outer membrane permeabilization and mitochondrial degradation (Pereira et al., 2007, 2010). As documented for the *aac3*Δ mutant, the deletion of the ADP/ATP carrier did not prevent the accumulation of ROS during the post-diauxic phase in cells expressing wtDFNA5 or mutDFNA5 (Figure 5C). However, the lack of Aac3 clearly interfered with the appearance of cell death markers. In the *aac3*Δ control cultures, the levels of dying cells were markedly higher as compared to control culture of wild-type cells, which is consistent with the fact that the *aac3*Δ mutant grows slower. The cultures with cells expressing wtDFNA5 or mutDFNA5 still displayed altered ratios between early or late apoptotic and necrotic cells, but the total number of cells with signs of cell death did not alter in the different growth phases. Furthermore, the total number of dying cells was comparable for the control culture and the culture of cells expressing wtDFNA5 and it was consistently lower for the culture of cells expressing mutDFNA5 (Figure 5D). Similar data were obtained for the *aac1*Δ mutant (data not shown). These observations confirm that mutDFNA5 requires the ADP/ATP carriers to instigate cytotoxicity and cell death. Furthermore, while ROS production has been described as an event common to most of the yeast apoptosis scenarios, our data demonstrate that in the ADP/ATP carrier mutants the correlation between ROS accumulation and viability does not hold. As such, our data are completely in line with previously reported results obtained with a triple *aac1-3*Δ mutant for acetic acid-induced apoptosis (Pereira et al., 2007).

Analysis of *aac3*Δ cells co-transformed with Mito-RFP and the EGFP-fusions revealed that these cells harbor a well developed mitochondrial tubular network. Even in cells expressing mutDFNA5-EGFP such a tubular network was present, but there were still punctuated mitochondria co-localizing with the foci of the EGFP fusion (Figure 5E). This led us to conclude that the absence of the ADP/ATP carriers did not prevent mutDFNA5 to interfere with mitochondrial fission and fusion dynamics or the clearance of fragmented mitochondria, which both are aspects that remain to be studied in more detail.

Finally, it should be noted that we did not observe significant differences in expression or degradation of the HA-tagged wtDFNA5 or mutDFNA5 proteins between the wild-type strain and the different mutant strains (Figure 5F). This indicates that the observed changes in DFNA5-instigated cytotoxicity in the mutant strains are related to their deleted functions and not to alterations in DFNA5 expression.

### DFNA5 toxicity is confined to its first globular domain

**Figure 6:**
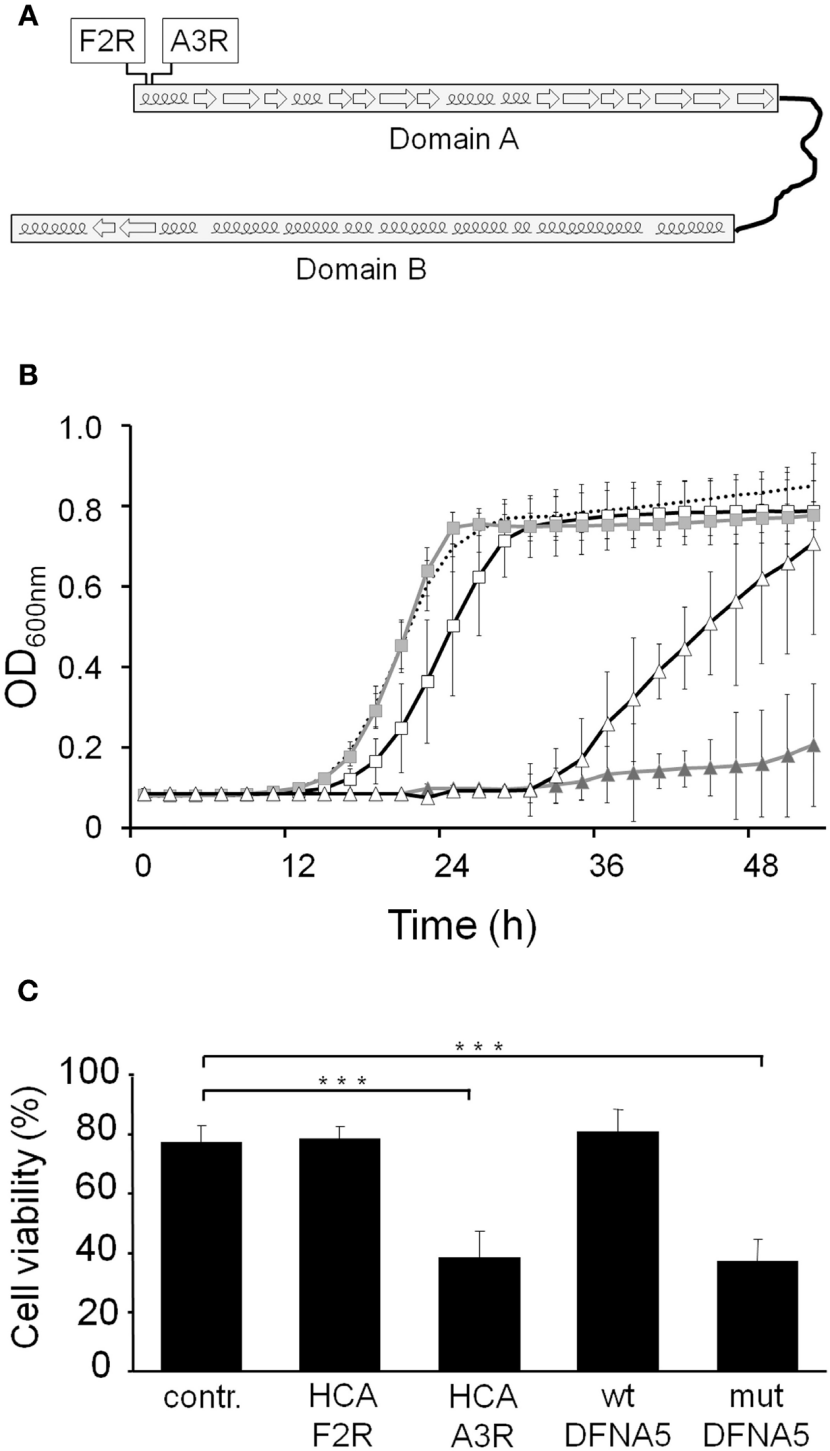
**DFNA5 cytotoxicity is confined to its first globular domain. (A)** Schematic representation of wtDFNA5 with the two globular domains connected by the hinge region and the indication of the mutants HCA-F2R and HCA-A3R (boxed) in the first domain. **(B)** Growth profiles of BY4741 cells expressing the first (gray triangles) or the second (gray squares) globular domain of wtDFNA5 or expressing point mutants in the first globular domain, i.e., mutants HCA-F2R (open squares) or HCA-A3R (open triangles). The dashed line is given as reference and depicts the growth of BY4741 cells transformed with an empty plasmid. **(C)** Cell viability of HEK293T cells transiently expressing the HCA-F2R and HCA-A3R mutants, wtDFNA5 and mutDFNA5 as indicated. Significances were assayed using One-Way ANOVA (^***^ = *p* < 0.001). Cells expressing EGFP served as control (contr.).

We recently proposed that wtDFNA5 is composed of two globular domains, which are separated by a hinge region (Figure 6A). In that study, we also demonstrated that the first domain induces apoptotic cell death in transfected HEK293T cells, which led to a model where the second domain can fold back to mask and regulate the apoptotic activity of the first domain (Op de Beeck et al., 2011b). Here, we expressed the two domains separately in yeast. As shown, the expression of the first domain, designated domain A and corresponding to the amino acid residues 1–256, triggered a very pronounced growth defect that by far surpassed the defect observed for mutDFNA5 (Figure 6B). Expression of the second domain, referred to as domain B and corresponding to residues 282–496, did not affect growth and the growth curve almost perfectly overlapped with the one obtained for the empty vector control.

DFNA5 belongs to the gasdermin protein family, named after the founder protein GSDMA, which is involved in gastric cancer and also harbors pro-apoptotic activities (Saeki et al., 2007). Sequence alignment of the different gasdermin family members show a high degree of conservation, especially in the first domain, which based on hydrophobic cluster analysis contains short α-helical folds interspaced by β-sheets (Op de Beeck et al., 2011b). To document the importance of these structures for the apoptotic-inducing activity of the domain, we used PCR-based site directed mutagenesis and phenotypically tested two of the mutants generated in domain A (Figure 6A). The first mutant, designated HCA-F2R, contained the substitution of a highly conserved hydrophobic phenylalanine into a basic arginine, thereby disrupting the first α-helical fold. When expressed in yeast, this mutant only triggered a small growth defect (Δ T1/2: 3.33 h ± 0.93) and thus lost most of the apoptotic-inducing activity (Figure 6B). In the second mutant, i.e., HCA-A3R, a non-conserved alanine residue was changed into arginine. Although this mutation also affected the first α-helical fold, its repercussions on the apoptotic-inducing activity were less pronounced as evidenced by the observation that the expression of this mutant in yeast resulted in an intermediate growth defect (Δ T1/2: 20.50 h ± 5.96; Figure 6B).

Next to the experiments in yeast, we also assessed the cell viability of human HEK293T cells transiently expressing the generated DFNA5 mutants. The expression of the HCA-A3R construct led to a significant decrease of cell viability (mean viability: 38.28% ± 9.37), whereas expression of HCA-F2R did not (mean viability: 78.60% ± 4.03), as it gave a similar cell viability as that observed for cells transfected with an empty EGFP vector (mean viability: 77.59% ± 5.58; Figure 6C). In fact, the cell viability of cells expressing HCA-A3R is highly comparable to those expressing mutDFNA5, while cell viability of cells expressing HCA-F2R is comparable to cells expressing wtDFNA5. As such, the results obtained in HEK293T cells confirm those obtained in yeast.

## Discussion

In this study we analysed the repercussion of heterologous expression of human wtDFNA5 or mutDFNA5 in *Saccharomyces cerevisiae*. Our data clearly demonstrate that mutDFNA5 causes a significant growth defect, which is associated with an increased number of late apoptotic and necrotic cells especially when the culture entered the stationary phase, and this in contrast to wtDFNA5. These findings confirm previous reported results showing that mutDFNA5 induces apoptotic and necrotic cell death when expressed in mammalian cells (Van Laer et al., 2004; Op de Beeck et al., 2011b).

Detailed analysis of the expression of wtDFNA5 and mutDFNA5 in the yeast system revealed that both proteins form inclusions. For wtDFNA5, these inclusions most likely correspond to IPOD as they are found at the periphery of the vacuole. The IPOD is a protein quality control compartment where proteins are deposited that presumably await autophagic clearance (Bagola and Sommer, 2008; Kaganovich et al., 2008). This suggests that wtDFNA5 is subjected to the normal cellular repertoire of protein quality control and clearance mechanisms. MutDFNA5 seems to escape these quality control systems. It does not form large IPOD-like deposits but rather smaller and more numerous foci, which are less intense and occurring mostly in the vicinity of the plasma membrane. Previous studies already suggested an association of mutDFNA5 with the plasma membrane and/or a membrane protein in mammalian cells, but the exact location remained unclear (Van Laer et al., 2004; Op de Beeck et al., 2011a). We now show that in yeast the foci formed by mutDFNA5 often co-localize with fragmented mitochondria, suggesting that the mutant proteins may interact either with the mitochondrial membrane or one of the mitochondrial membrane proteins, which in turn may lead to mitochondrial impairment. The latter is further evidenced by the fact that mutDFNA5 is even more toxic in the *fis1*Δ and *por1*Δ mutants, while it lost its specific toxicity-inducing capacity in mutant strains lacking the ADP/ATP carriers Aac1 and Aac3. It is remarkable that the same mitochondrial proteins have previously been identified as key players with similar contributions for acetic acid-induced apoptosis in yeast (Fannjiang et al., 2004; Pereira et al., 2007). This underscores that mutDFNA5-instigated cytotoxicity and acetic acid-induced apoptosis build on common molecular mechanisms. Note that we did not analyse the third ADP/ATP carrier Aac2 in our studies for the simple reason that its deletion is lethal in the BY4741 background (Chen 2004).

The studies on acetic acid-induced apoptosis demonstrated a protective function of Fis1 that relates to inhibition of Dnm1-mediated mitochondrial fission and possible additional pro-apoptotic Dnm1 functions (Fannjiang et al., 2004). Also for mutDFNA5-induced cell death we found that Fis1 fulfils a protective role but this apparently does not involve Dnm1 or its adaptor Mdv1. Indeed, our observation that neither the deletion of *DNM1* nor of *MDV1* prevented mutDFNA5-induced cell death excludes these fission proteins as downstream effectors. That Fis1 could have a specific function not shared by the other fission factors Dnm1 and Mdv1 was already noted before in studies dealing with ethanol-induced apoptosis. These studies suggested that Fis1 has a specific role for the maintenance of mitochondrial fragmentation in response to ethanol (Kitagaki et al., 2007). However, most recent studies revealed that *fis1*Δ mutants accumulate secondary loss-of-function mutations in the *WHI2* gene (Cheng et al., 2008; Mendl et al., 2011), which encodes a protein involved in cell cycle regulation (Radcliffe et al., 1997), the general stress response, actin dynamics and Ras-cAMP-PKA signaling (Kaida et al., 2002; Leadsham et al., 2009) as well as the selective degradation of dysfunctional mitochondria via autophagy, a process known as mitophagy (Muller and Reichert, 2011). In fact, the studies on cell death and mitophagy showed that the enhanced sensitivity of the *fis1*Δ mutants toward cell death stimuli is solely due to the loss of the Whi2 function and not to the lack of Fis1 (Cheng et al., 2008; Mendl et al., 2011). Also the *fis1*Δ mutant strain of the yeast deletion collection that was used in our study appears to contain such a secondary loss-of-function mutation in *WHI2* (Cheng et al., 2008). Hence, it is feasible that the enhanced mutDFNA5-instigated cytotoxicity in the *fis1*Δ mutant strain relates to the *WHI2* mutation and the consequent diminished stress resistance and lower rate of mitophagy, rather than to a deficiency in mitochondrial fission. At least, it would explain the observed accumulation of fragmented and aggregated mitochondria co-localizing with the foci of mutDFNA5-EGFP in the *fis1*Δ mutant, and as such provide an additional confirmation that mutDFNA5 triggers cell death through mitochondrial damage.

Another aspect of Fis1 is that the protein has similar biophysical properties as the mammalian Bcl2 and Bcl-xL and although these anti-apoptotic proteins cannot replace the mitochondrial fission function of Fis1, they do substitute for Fis1 in cell viability assays (Fannjiang et al., 2004). Bcl2 and Bcl-xL have important roles as regulators of mitochondrial membrane permeabilization, since they inhibit non-specific pore formation by the adenine nucleotide translocator, ANT, the mammalian ortholog of the Aac1/2/3 ADP/ATP carriers (Brenner et al., 2000; Belzacq et al., 2003). Previous studies in yeast identified Fis1 as a potential regulator together with the mitochondrial permeability transition pore components Aac1/3 and the VDAC protein Por1 for acetic acid-induced cell death (Fannjiang et al., 2004; Pereira et al., 2007, 2010). Our data now demonstrate that mutDFNA5 cytotoxicity is enhanced in the absence of Por1, while this toxicity is basically abrogated in the absence of Aac1 or Aac3. Whether this means that mutDFNA5 directly targets the ADP/ATP carriers to alter mitochondrial membrane permeability remains to be clarified. In humans, mutations in the *ANT1* gene are associated with progressive external ophthalmoplegia (Sharer, 2005) and to our knowledge there are no reports that link *DFNA5* to this disorder or, conversely, that link *ANT1* to autosomal dominant deafness.

DFNA5 belongs to the gasdermin family. Although the members of this family appear to have different molecular functions, they share conserved structural features, such as the presence of a globular domain in their N-terminal half (Saeki et al., 2007; Tamura et al., 2007; Op de Beeck et al., 2011b). Intriguingly, this domain harbors the DFNA5 capacity to induce cell death as confirmed in previous studies (Op de Beeck et al., 2011b) and ours. It is not known whether this capacity to induce cell death is a common physiological property of all gasdermin family members, but at least for one other member, i.e., GSDMA, this seems to be the case since the protein was reported to induce apoptosis in a gastric epithelial cell line (Saeki et al., 2007). The apoptosis-inducing globular domain was proposed to be shielded in wtDFNA5 by a second C-terminal regulatory domain. In mutDFNA5 a large part of this regulatory domain is missing and therefore the apoptosis-inducing domain is presumably more exposed (Op de Beeck et al., 2011a,b). Structurally, the apoptosis-inducing domain is composed of short α-helical folds interspaced by β-sheets. Here, we show that mutations disrupting the first α-helical fold strongly reduce the cell death-inducing capacity of the N-terminal domain in yeast and human HEK293T cells. This demonstrates the feasibility to use the yeast system to further dissect the structural requirements of DFNA5 associated with its apoptosis-inducing property.

### Conflict of interest statement

All authors declare that the research was conducted in the absence of any commercial or financial relationships that could be construed as conflict of interest. The funders had no role in study design, data collection and analysis, decision to publish, or preparation of the manuscript. Joris Winderickx declares that he is co-founder of the KU Leuven spin-off companies reMYND and ADxNeuroSciences, but this did not influence study design, data collection, analysis, publication, or the preparation of the manuscript.
